# Implementing Anti‐Amyloid Therapies in Memory Clinics: A Qualitative Study of Clinician Experiences and Practice Changes

**DOI:** 10.1002/alz70861_108594

**Published:** 2025-12-23

**Authors:** Ayush Thacker, Anna L Parks, Daniel Dohan, Seth A Gale, Liliana Ramirez Gomez, Christine S Ritchie, Sachin J Shah, Joanna Paladino

**Affiliations:** ^1^ Mongan Institute Center for Aging and Serious Illness, Division of Palliative Care and Geriatric Medicine, Massachusetts General Hospital, Boston, MA USA; ^2^ University of Utah, Salt Lake City, UT USA; ^3^ University of California, San Francisco, San Francisco, CA USA; ^4^ Center for Brain/Mind Medicine, Brigham and Women's Hospital, Boston, MA USA; ^5^ Massachusetts General Hospital, Harvard Medical School, Boston, MA USA; ^6^ Mongan Institute Center for Aging and Serious Illness, Division of General Internal Medicine, Massachusetts General Hospital, Boston, MA USA

## Abstract

**Introduction:**

Anti‐amyloid therapies (AATs) have changed the diagnosis and treatment paradigm of Alzheimer’s disease (AD). Our interdisciplinary team completed a qualitative study of clinicians’ experience with AAT implementation.

**Methods:**

Our interdisciplinary team conducted a qualitative study examining clinician experiences with AAT implementation across seven U.S. academic medical centers with representation from all regions across the United States. We conducted semi‐structured interviews with 27 prescribing clinicians and analyzed transcripts using thematic content analysis.

**Results:**

Our findings highlight three major themes. First, AATs affect practice norms for AD diagnosis. Clinicians feel added pressure for time‐efficiency of the diagnostic process, sense expectations for more accurate diagnoses, and integrate AAT considerations into diagnostic disclosure and choice of tests. Even with increased time pressure, conversations about AATs are unfolding over multiple visits because of the time it takes for eligibility tests to return and to allow for shared decision‐making. Second, AATs represent a paradigm shift that brings both promise and complexity to dementia care. Clinicians expressed renewed hope in treatment options, which they described as an "antidote to nihilism" in dementia care. However, this optimism is tempered by increased workload, acute management pressures, and the challenge of maintaining quality care for non‐AAT patients amidst shifting priorities and limited institutional support. Third, clinicians and institutions vary in lecanemab treatment protocols, including comfort with offering lecanemab to patients on concurrent anticoagulants or homozygous for ApoE4. While institutional protocols initially adhered closely to published appropriate use criteria regarding patient eligibility, clinicians reported modifications of eligibility criteria over time.

**Conclusions:**

Clinicians and clinical leaders can use findings from this study to consider structural changes to accommodate new workflows related to AAT and to inform the development of AAT decision‐support interventions.

